# Effects of Evaporation and Body Thermal Plume on Cough Droplet Dispersion and Exposure Risk for Queuing People

**DOI:** 10.3390/life15010028

**Published:** 2024-12-30

**Authors:** Fengjiao Li, Guoyi Jiang, Fei Chen, Weibin Yuan

**Affiliations:** 1 Department of Civil Engineering, Zhejiang University of Technology, Hangzhou 310012, China; lifengjiao@zjut.edu.cn (F.L.);; 2 Department of Civil Engineering and Smart Cities, Shantou University, Shantou 515063, China

**Keywords:** droplet dispersion, cough, virtual manikin, computational fluid dynamics (CFD), outdoor environment, exposure risk, evaporation

## Abstract

The transmission of virus-containing droplets among multiple people in an outdoor environment is seldom evaluated. In this study, an Euler–Lagrange computational fluid dynamics approach was used to investigate the effects of evaporation and the body thermal plume on the dispersion of coughed droplets under various wind conditions, and the infection risk was evaluated for various arrangements of individuals queuing outdoors using virtual manikin models. The evaporation time was longer for larger droplets and in a more humid environment. Transient evaporation strongly affected the motion of droplets ranging in diameter from 60 to 150 μm. The body thermal plume affected airflow and particle dispersion under weak wind conditions, but its effect was negligible at wind speeds greater than 0.8 m/s. Droplets smaller than 100 μm could reach the head of a susceptible person, suggesting a high exposure risk. The exposure fraction and body deposition were highest in an all-male queue sequence and lowest for a male–female–male–female–male queue sequence.

## 1. Introduction

In 2019, the novel coronavirus disease (COVID-19) rapidly spread around the world, causing great damage to society, human health, and the global economy. COVID-19 has not been eradicated, and other respiratory diseases, including type A, B, and C influenza, are also frequently epidemic. These infectious viruses are mainly transmitted by respiratory droplets [1]. To develop measures for reducing infection, studies are necessary to evaluate the dispersion of virus-containing droplets and assess the risk of exposure in different living environments. Many scholars have conducted extensive work to understand the transmission characteristics of respiratory droplets and the exposure risk of infectious viruses under various flow conditions.

Studies on the dispersion of respiratory droplets began in the 19th century. Researchers used glass slides and oral staining agents to determine the size distribution of droplets generated by various respiratory activities [2]. Wells [3] analyzed the pattern of respiratory particle transmission in 1934. The development of novel microscale techniques and imaging technologies has enabled more comprehensive explorations of the diameter distributions of expelled droplets [4,5,6] and of exhaled airflow velocities [7,8,9]. These explorations have provided detailed initial data regarding respiratory activities and accurate boundary conditions for further numerical simulation studies on particle transport. Computational fluid dynamics (CFD) models based on an Euler–Lagrange method have been validated and are widely used to simulate the transmission of airborne particles [10,11]. Most studies have focused on the dispersion of respiratory droplets in enclosed environments, such as aircraft cabins [12,13,14,15], high-speed rail cabins [16], elevators [17], and ventilated rooms [18,19,20,21]. Regarding enclosed indoor environments, Salmanzadeh et al. [22] and Laverge et al. [23] investigated the influence of the body’s thermal plume on droplet dispersion and found that the thermal plume greatly affects the shape of the breathing zone and the concentration distributions of particles suspended in this zone.

Recently, studies on the transmission of respiratory droplets have been extended to evaluate the exposure risk in an outdoor environment. Li et al. [24] investigated the influence of wind direction on the dispersion of coughed droplets around an isolated human body and found that, when a person stood with his back to the wind, the droplets spread widely and traveled slowly because of the reverse flow downstream, increasing exposure time and infection risk. In an outdoor environment, wind speed and social distance are the factors that most strongly affect droplet dispersion and therefore exposure risk. Dbouk and Drikakis [25] computationally investigated the effect of outdoor wind speed on particle transport and found that saliva droplets can travel up to 6 m when wind speeds are 4–15 km/h. To evaluate the exposure risk posed by respiratory droplets, several studies have investigated the combined effects of wind speed, relative humidity, and social distance on the dispersion and deposition of respiratory droplets for two persons standing face-to-face outdoors [26,27,28,29]. In these studies, the wind speeds were 0.2–5.5 m/s, and the social distances were 0.5 to 5.0 m. The adopted manikin models had either simplified geometry [26,28] or a realistic human body [27,29]. The distance travelled by a small particle was found to be insensitive to the relative humidity, and medium-sized particles (>60 μm) were the size of particles most likely to be deposited. Although increasing social distance is typically recommended, its effect on infection risk was ambiguous; standing at an angle to the prevailing wind direction was suggested as a means of decreasing risk. Jiang et al. [29] strongly recommended using realistic human body geometry in outdoor simulations because aerodynamic effects caused by the body′s shape can greatly affect flow and particle dispersion.

In an outdoor environment, most articles focused on the dispersion of respiratory droplets around a single-manikin model or two face-to-face persons; the propagation and transmission of virus-containing droplets among multiple people have seldom been investigated. However, outdoor queuing is common in society, both during typical activities such as shopping and activities with a high infection risk, such as in hospitals or during COVID-19 screenings. Therefore, evaluating the exposure risk in such scenarios is critical for helping policymakers prevent the spread of respiratory diseases and reduce healthy people’s risk of infection. Moreover, the influences of evaporation and the human thermal plume on the transport of respiratory droplets should be further studied in an outdoor environment. This study focused on the dispersion of coughed droplets in an outdoor environment using an Euler-Lagrange model of CFD. The objectives of this study were to investigate the impact of evaporation on the motion of droplets through empty space, to study the influence of the human thermal plume on droplet dispersion under different wind conditions by using a single-manikin model, and to evaluate the exposure risk posed by cough activity for people standing in various multi-person models. Infection risk was evaluated in terms of particle depositions on the human body and the exposure fractions in the breathing zone. The different dispersion patterns of droplets in the single-manikin model and multi-person model were also compared. This study has practical significance for preventing and controlling the spread of respiratory diseases. The structure of this study is as follows. Section 1 summarized the literature review about the transmission of respiratory droplets in indoor and outdoor environments. Section 2 introduced the theories about droplet dispersion and evaporation. Detailed information about simulation settings and boundary conditions was explained in Section 3. Section 4 presented the results and discussions. Finally, the conclusions and limitations were given at the end of the text.

## 2. Droplet Transport and Evaporation Theories

### 2.1. Discrete-Phase Motion Model

This study applied an Euler–Lagrange approach to simulate the wind flow and particle dispersion. Air was considered to constitute a continuous phase, and the Euler approach was used to simulate the wind flow. Assuming that the flow was incompressible, unsteady Reynolds-averaged Navier-Stokes (RANS) simulations were conducted using a renormalization group (RNG) *k*-*ε* model [30]. The governing equations for the continuous phase were the continuity, momentum, temperature, and transport equations of the turbulent kinetic energy *k* and turbulent dissipation rate *ε*. The RNG model was adopted because it has been fully validated and is widely used to simulate blunt-body flow in the wind engineering field.

This study considered droplets as a discrete phase, and the Lagrange approach was used to simulate the particle dispersion. The discrete-phase model was selected because of its applicability for studying particle diffusion when the volume fraction of droplets is much lower than that of the continuous phase. The discrete-phase equation of motion was solved by tracking the trajectories of numerous particles in accordance with Newton’s second law as follows:

(1) $$m_{p}\frac{d{\vec{u}}_{p}}{dt}={\vec{F}}_{D}+{\vec{F}}_{G}+{\vec{F}}_{B}+{\vec{F}}_{S}$$

where *m*_*p*_ = *πρ_p_*$d_{p}^{3}$/6 is the mass of a droplet, ${\overset{⃑}{u}}_{p}$ is the velocity of a particle in vector form, and *ρ*_*p*_ and *d_p_* are the density and diameter of a particle, respectively. The terms on the right side of the equation correspond to all of the forces exerted on a droplet by the fluid phase. These forces are the drag force ${\overset{⃑}{F}}_{D}$, the gravity force ${\overset{⃑}{F}}_{G}$, the buoyant force ${\overset{⃑}{F}}_{B}$, and other forces ${\overset{⃑}{F}}_{S}$. If the droplets are considered to be spherical, a spherical drag law can be used to calculate the drag force as follows:

(2) $${\vec{F}}_{D}=-\frac{1}{8}\pi C_{D}\rho d_{p}^{2}\times \left|{\vec{u}}_{P}-\vec{u}\right|\left({\vec{u}}_{p}-\vec{u}\right)$$

in which *C_D_* is the drag coefficient, *ρ* is the density of the air phase, and $\overset{⃑}{u}$ is the vector-form velocity of the fluid phase. The buoyant force can be calculated using Archimedes’s law as follows:

(3) $${\vec{F}}_{B}=-\frac{\rho }{\rho_{p}}m_{p}\vec{g}$$

The other forces exerted on the droplets are the virtual mass force, pressure gradient force, Brownian force, and Saffman lift force. These additional forces were ignored in this study because the virtual mass and pressure gradient forces are negligible when the density of the fluid is much lower than that of the particles, and the Brownian and Saffman’s lift forces are non-negligible for only sub-micron particles.

The influence of wind turbulence on particle dispersion was included by using a stochastic tracking model; in the model, the three velocity fluctuations were obtained as follows;

(4) $$u_{i}^{\prime }=\zeta \sqrt{2k/3}$$

where *ζ* is a normally distributed random number and *k* is the turbulent kinetic energy.

### 2.2. Droplet Evaporation Model

The mass of a droplet *m_p_* changes over time through evaporation. The evaporation process is controlled by the following mass transfer rate equation:

(5) $$\frac{dm_{p}}{dt}=-\pi d_{p}D_{dyn}Sh\frac{M_{v}}{M_{m}}\ln\frac{P-P_{v,s}}{P-P_{v,m}}$$

where *D_dyn_* is the dynamic diffusivity of water vapor in the continuum; *Sh* is the Sherwood number; *M_v_* and *M_m_* are the molecular weights of the vapor and mixed air, respectively; *P* is the pressure of ambient air; and *P_v_*_,*s*_ and *P_v_*_,*m*_ are the partial pressures of water vapor at the droplet surface and in the humid air, respectively.

The decrease in droplet′ temperature because of the latent heat of phase change was calculated by:

(6) $$m_{p}c_{p,p}\frac{dT_{p}}{dt}=q_{m,p}-h_{lv}\frac{dm_{p}}{dt}$$

where *c_p_*_,*p*_ and *h_lv_* are the heat capacity and latent heat of water; *q_m_*_,*p*_ is the interfacial heat transfer rate; *T_p_* is the temperature of droplets.

### 2.3. Thermal Buoyancy Theory

The influence of the human body’s thermal plume on particle dispersion was investigated; the Boussinesq assumption was used to simplify the solved equations. In the model, the air density was treated as constant in all solved equations except for the buoyancy term in the momentum equation; this is accurate if changes in actual density and temperature are small. For most natural-convection flows, convergence can be accelerated by adopting the Boussinesq assumption. In a Boussinesq model, the density in the momentum equation is obtained as follows:

(7) $$\rho_{b}\approx \rho [1-\beta (T-T_{0})]$$

where *ρ* is the air density, *β* is the thermal expansion rate, *T* is the air temperature, and *T*_0_ is the reference temperature.

## 3. Simulation Settings and Boundary Conditions

### 3.1. Manikin Model and Cough Activity

The virtual manikins were adult male and female models in a standing posture. To capture the detailed airflow and particle dispersion characteristics around a human body, near-real manikin models designed by the ITO Laboratory [31] were modified such that their shapes matched the main geometric parameters of East Asian individuals. The body height *H* was set to 1.74 m for the male model and 1.60 m for the female model. Detailed features, such as hair shape were also considered for the female model. More information regarding the adopted manikin models can be found in [29].

Coughing was selected as the respiratory activity because it is one of the most common activities that produces numerous droplets and spreads airborne disease. Many experiments on cough activity have been conducted [5,7,32] and have obtained detailed cough information, such as the velocity of the jet from the mouth and droplet numbers, for use as parameters in a numerical simulation. In this study, the value of the transient volumetric flow rate for a single cough was set to that in the experimental results of Gupta et al. [7]. In their experiment, the duration of cough activity was 0.5 s, the peak airflow rate was approximately 4.8 L/s, and this peak occurred 0.1 s after coughing. The number and sizes of exhaled droplets were set in accordance with the experiments performed by Duguid [2], which were reproduced by Bourouiba et al. [32]. A total of 4973 droplets were considered to be exhaled from one cough, and the size of the droplets ranged from several micrometers to thousands of micrometers. The majority of droplets were of diameter 8, 16, or 24 μm. A droplet comprised 98.2% water and 1.8% droplet nucleus. Because the micron-droplets covered a wide range in Duguid’s distribution data, it has the benefit of observing the diffusion patterns of droplets of different sizes. However, this distribution data overestimated the original diameters of coughing droplets because of the restriction of the measuring techniques compared to new micro-techniques and imaging technologies nowadays [5].

### 3.2. Simulation Cases and Boundary Conditions

**Figure 1:**
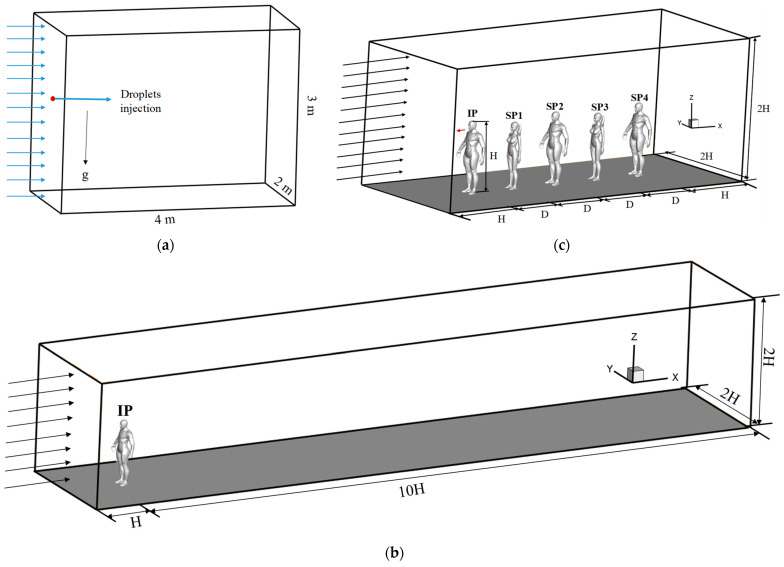
Geometric models and computational domains: (**a**) empty domain; (**b**) single-manikin model; (**c**) multi-person model.

To investigate the effect of evaporation on droplet dispersion, two cases with different relative humidity (RH) were investigated (RH = 0% and 90%) in an empty domain with a size of 4 m × 2 m × 3 m [Figure 1a]. A total of 125 droplets with a diameter of 2–250 μm were injected into the domain from the inlet boundary at a point 1.55 m above the ground in each simulation. A small wind velocity of 0.25 m/s was set for the inlet boundary (the arrows from the inlet indicated the wind direction), and the trajectory of each particle under the influence of the inlet wind was recorded. To validate the droplet evaporation model, the evaporation results for droplets with a diameter of 10 or 100 μm were compared with theoretical results calculated by Wei and Li [33].

To examine the influence of the body thermal plume on the dispersion of coughed droplets, eight simulations were conducted using a single-manikin model with and without considering the body thermal plume under four inflow wind speeds *V_in_*: 0.2, 0.4, 0.6, and 0.8 m/s. Droplet evaporation was considered, and the humidity of the environment was set to 60%. In accordance with the findings of Mamdud et al. [34] for an adult in a standing posture, the heat flux generated by the human body was set to 57 J/(m^2^∙s) in this study. Figure 1b displays the computational domain for the single-manikin model. An isolated male model stood facing the wind. A long domain of 11*H* was used in the streamwise direction to sufficiently capture the droplet dispersion.

Three cases were conducted to study the dispersion of cough droplets around multiple people when considering droplet evaporation (RH = 60%) but without considering the body thermal plume. An inflow wind speed of *V_in_* = 1.8 m/s was adopted because this is a typical wind speed in outdoor environments. Three five-person queues were modeled with different sex arrangements: five men (TQ-1), male–female–male–female–male (TQ-2), and female–male–female–male–female (TQ-3). Figure 1c displays the arrangement for the second queuing type. Five people stand in the computational domain facing the oncoming wind at a 1 m distance from each other. The first person is an infected person (IP) and generates respiratory droplets by coughing. The people standing downstream are susceptible people (SPs), denoted SP1, SP2, SP3, and SP4, respectively. A short distance behind SP4 in the streamwise direction was used, decreasing the domain length and reducing the mesh number; infection risk was the main research objective, and modeling droplets that had passed the last person was unnecessary.

**Figure 2:**
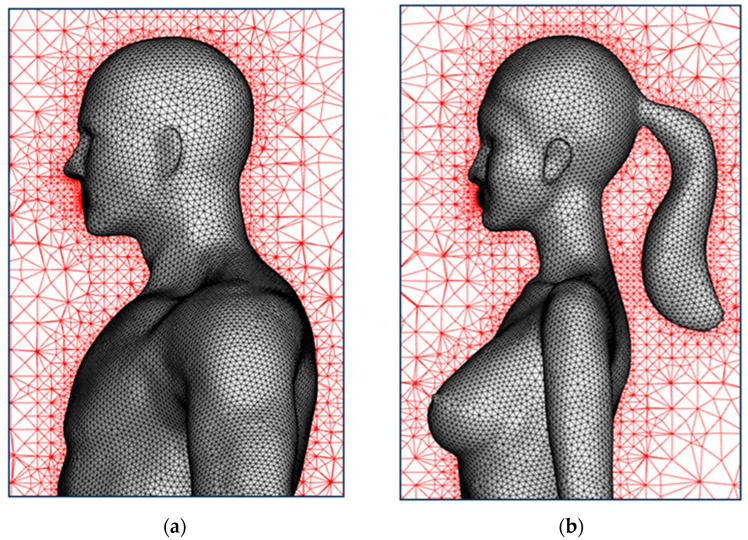
Mesh distribution around the manikins of the: (**a**) male model; (**b**) female model.

For all simulations, the top and two side boundaries of the domain were set to symmetrical conditions, and a wall condition was set for the ground and the surfaces of the bodies. For droplets, a “trap” condition was used for the wall and an “escape” condition was adopted for the outlet boundary; that is, the tracking of a particle was stopped when the particle reached a wall or an outlet boundary. An unstructured mesh system with a cell number of 6,500,000 was generated for both the single-manikin model and multiperson models. The maximum size of the mesh was controlled to be less than 8 mm for the human body surface, ensuring that airflow and particle dispersion around the manikin were accurately captured. The maximum mesh was reduced to less than 2 mm for the mouth because the coughed droplets were injected into the domain from the mouth. Figure 2 displays the mesh distributions around the manikin models in detail.

The RNG turbulence model introduced in Section 2 was adopted for the simulations. The SIMPLE algorithm was employed for pressure–velocity calculations. A second-order Upwind scheme, which is commonly used in wind engineering, was adopted to discretize the convection terms. A fully developed flow field was taken as the initial conditions for the transient simulations, in which the motions of droplets were tracked. A time step of Δ*t* = 0.005 s was adopted to ensure the stability of the transient simulations. The simulations were stopped when almost no particles were detected in the domain.

## 4. Results and Discussion

### 4.1. Effects of Evaporation on Droplet Dispersion

**Figure 3:**
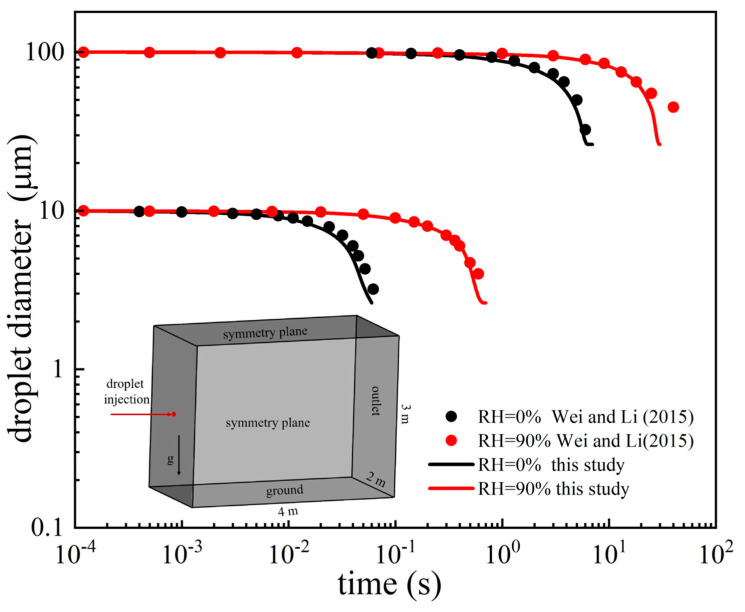
Validation of the evaporation model [33].

Wei and Li [33] provided an accurate theoretical model of the evaporation of dispersed droplets with diameters of 10 and 100 μm in an empty domain; their model was used to validate the evaporation model adopted in this study. Figure 3 compares the changes in diameter over time for the 10-μm and 100-μm particles at RH of 0% and 90% (black and red, respectively). Dots indicate the predictions of the theoretical model [33], and the lines are the CFD results obtained in this study. Nicas et al. [35] proved that the diameter of a droplet reaches 0.262 times its initial diameter after evaporation but then no longer changes when the surface temperature of the droplet equals to the environmental temperature. This is why the lines predicted by the CFD simulations in Figure 3 became horizontal after evaporation finished. The 10-μm droplet evaporated completely after 0.06 s at RH = 0% but after 6 s at RH = 90%. The 100-μm droplet required approximately 5.6 s to fully evaporate at RH = 0% but 30 s at RH = 90%. Thus, for droplets with a given diameter, evaporation requires a much longer time at a higher RH. Similarly, for a given RH, the complete evaporation time is longer for the larger droplet. The evaporation predicted by the numerical simulations was consistent with the theoretical calculations. Therefore, the droplet evaporation model adopted in this study was considered accurate and applicable for predicting droplet evaporation.

**Figure 4:**
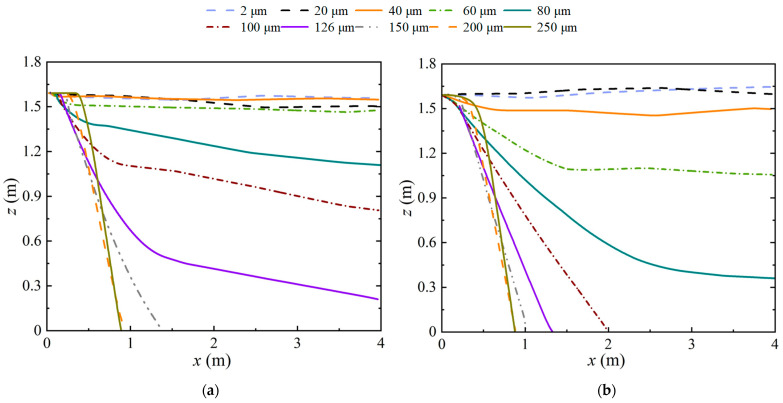
Trajectories of different-sized droplets under: (**a**) RH = 0%; (**b**) RH = 90%.

Figure 4 presents comparisons of the motion trajectories of 10 representative droplets with diameters of 2, 20, 40, 60, 80, 100, 126, 150, 200, and 250 μm at low humidity (RH = 0%) and high humidity (RH = 90%). Both droplet size and humidity affected the dispersion characteristics. The trajectories of droplets with diameters less than 40 μm were nearly horizontal and were insensitive to humidity. Droplets larger than 150 μm quickly dropped to the ground because of gravity; their trajectories were only slightly affected by the humidity. The trajectories of medium-sized particles were more sensitive to humidity because of evaporation. In dry air (RH = 0%), the trajectories of droplets with *d_p_* = 60–80 μm were near-horizontal because of the droplets’ rapid evaporation. However, at RH = 90%, they first moved downward at an incline and then moved nearly horizontally as their diameters decreased due to slow evaporation. Droplets with *d_p_* = 100 and 126 μm completed evaporation and left the domain when the air was dry, but they quickly landed without finishing evaporation in the humid environment. A droplet with a horizontal traveling distance of 4 m could reach the outlet boundary of the computational domain before landing. Thus, droplets with a diameter smaller than 126 μm could reach the outlet boundary of the domain in dry air, but only droplets smaller than 80 μm could reach the outlet when the environmental humidity was high because the moist air suppressed droplet evaporation.

### 4.2. Effect of Body Thermal Plume on Droplet Dispersion

**Figure 5:**
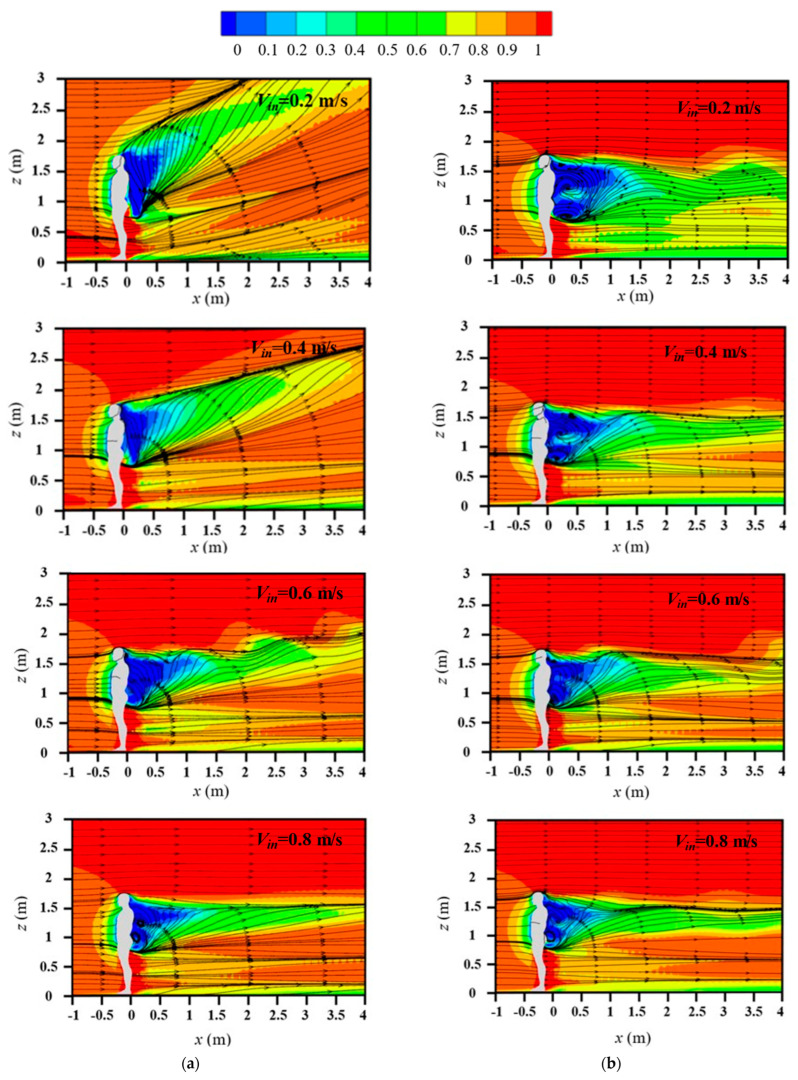
Streamlines and normalized streamwise velocity in the center plane: (**a**) considering body heat dissipation; (**b**) without considering body heat dissipation.

Figure 5 displays the streamlines and normalized streamwise velocity around an isolated human body in the center plane for various inflow wind speeds and with and without considering the body thermal plume. For cases without considering the body thermal plume (right column in Figure 5), a reverse and weak flow was found to form behind the body. The influence of wind speed on the flow field was small except for very weak inflow (*V_in_* = 0.2 m/s); in this case, the weak flow area behind the body was slightly larger. The body thermal plume had a strong influence on the airflow pattern around the body, especially for the weak inflow wind cases. For the weak wind flow cases of *V_in_* = 0.2 and 0.4 m/s, clear rising flow occurred behind the human body because of the influence of body thermal plume; this rising flow extended above the head. As the incoming wind speed increased, the influence of the body thermal plume on airflow decreased. When the inflow wind speed was 0.8 m/s, the body thermal plume was completely suppressed by the oncoming wind and had nearly no effect on the flow features. Thus, the effect of the body thermal plume should be considered when the inflow wind is weak but can be ignored if the inflow wind is larger than 0.8 m/s.

**Figure 6:**
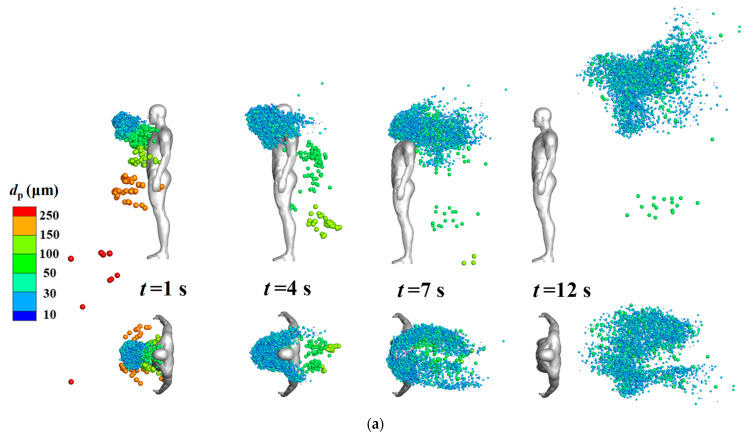

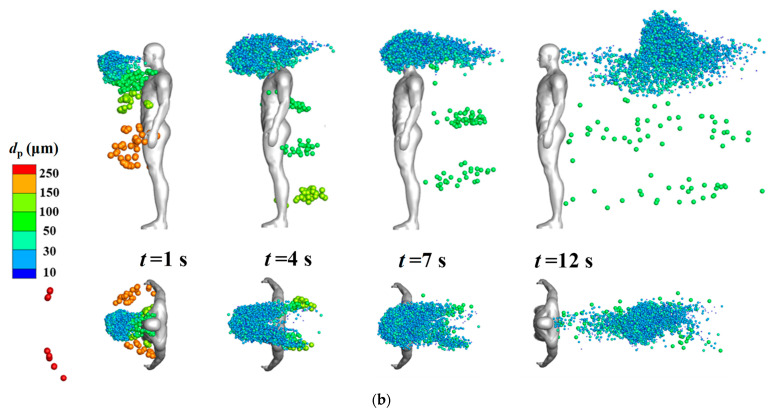
Droplet distribution around the isolated human model at various time points (*V_in_* = 0.2 m/s): (**a**) considering the body thermal plume; (**b**) without considering the body thermal plume.

**Figure 7:**
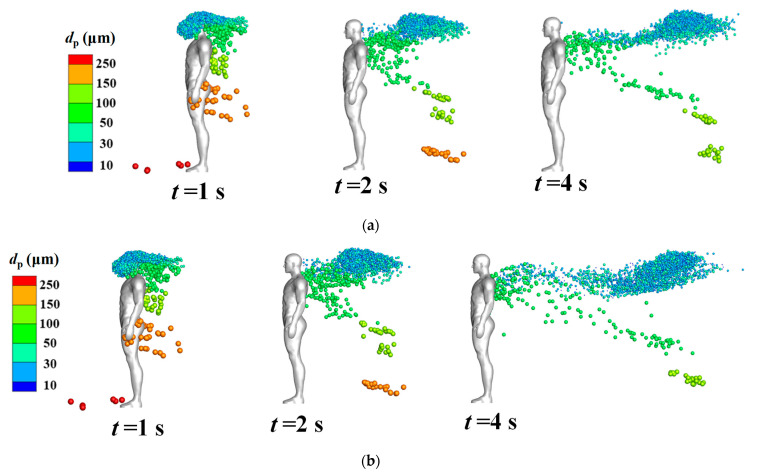
Droplet distribution around the isolated human model at various time points (*V_in_* = 0.8 m/s): (**a**) considering the body thermal plume; (**b**) without considering the body thermal plume.

Figure 6 displays the distribution of coughed droplets around an isolated human body at different time steps when the inflow wind was extremely low (*V_in_* = 0.2 m/s). The indicated sizes of all particles in the legend are their initial diameters, but the droplets’ diameters gradually decreased because of evaporation. Particle diameter affected the particle dispersion characteristics. In the cases without considering the body thermal plume [Figure 6b], droplets larger than 150 μm quickly dropped in front of the human body after they were injected from the mouth; medium particles (50–150 μm) gradually dropped behind the human body after they were blown downstream; and droplets smaller than 50 μm were first blown back toward the head position and then blown downstream horizontally to cluster in a narrow band. The body thermal plume greatly affected the diffusion of cough droplets when the environmental wind speed was low. At *t* = 12 s, the droplets smaller than 50 μm had been transported to a much higher position because of the rising flow caused by the body thermal plume. Compared with the cases without this thermal plume, the dispersion area of the small droplets in the horizontal direction was wider when the plume was considered. This was likely because the thermal plume increased turbulence. A slight asymmetry distribution of particles in *t* = 12 s in Figure 6a may come from the following two reasons. First, the flow field cannot become totally symmetry because of the flow disturbance caused by the complex body shape of the real manikin model, and the body thermal plume may also slightly destroy the symmetry of the flow. Second, the droplets were not uniformly injected into the flow field from the grid of the human mouth. After the droplets were discharged, the complex airflow around the human body further amplified these position differences during the propagation of droplets. Figure 7 displays the droplet distribution around the isolated human body at different times when the inflow wind was 0.8 m/s. Under this condition, the dispersion of the droplets was dominated by the environmental wind speed and the aerodynamic effect of the body shape; the influence of the body thermal plume on particle dispersion was nearly negligible.

**Figure 8:**
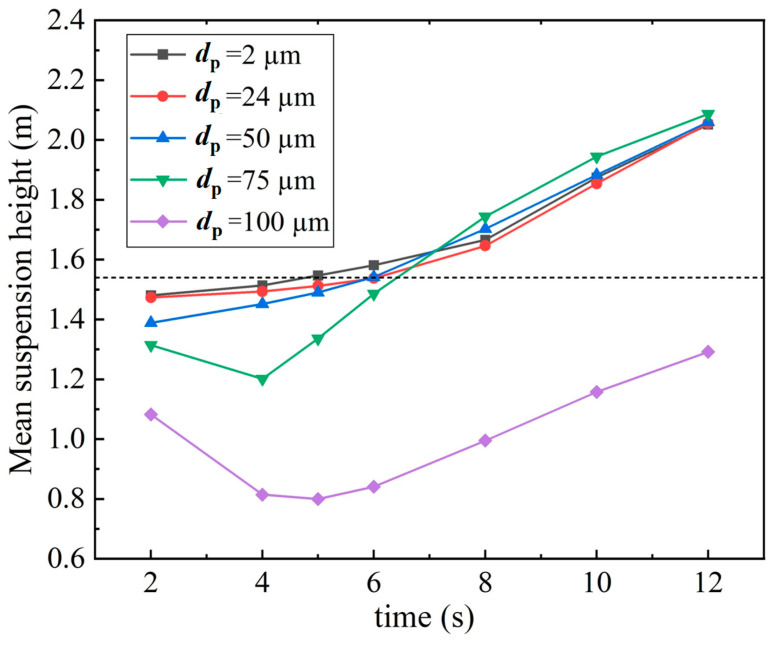
Mean suspension height for droplets smaller than 100 μm over time under low wind speed conditions and considering the body thermal plume (*V_in_* = 0.2 m/s).

The results revealed that droplets with an initial diameter of less than 100 μm exhibited diverse propagation characteristics. Figure 8 displays the average suspension heights of these particles for *V_in_* = 0.2 m/s to further illustrate the effects of evaporation and the body thermal plume on particle dispersion. The mean suspension heights for all droplets smaller than 50 μm were similar; they gradually increased over time because of the rising flow caused by the body thermal plume. For droplets with diameters of 75 and 100 μm, the mean suspension heights initially gradually decreased because gravity dominated their motion; after *t* = 4 s, the heights increased gradually because the particle sizes decreased due to evaporation and the droplets were affected by the rising flow generated by the thermal plume. Consequently, the dispersion of medium-sized particles was strongly affected by both evaporation and the body thermal plume under weak wind flow conditions.

### 4.3. Evaluation of Infection Risk for Standing People in the Multiperson Model

**Figure 9:**
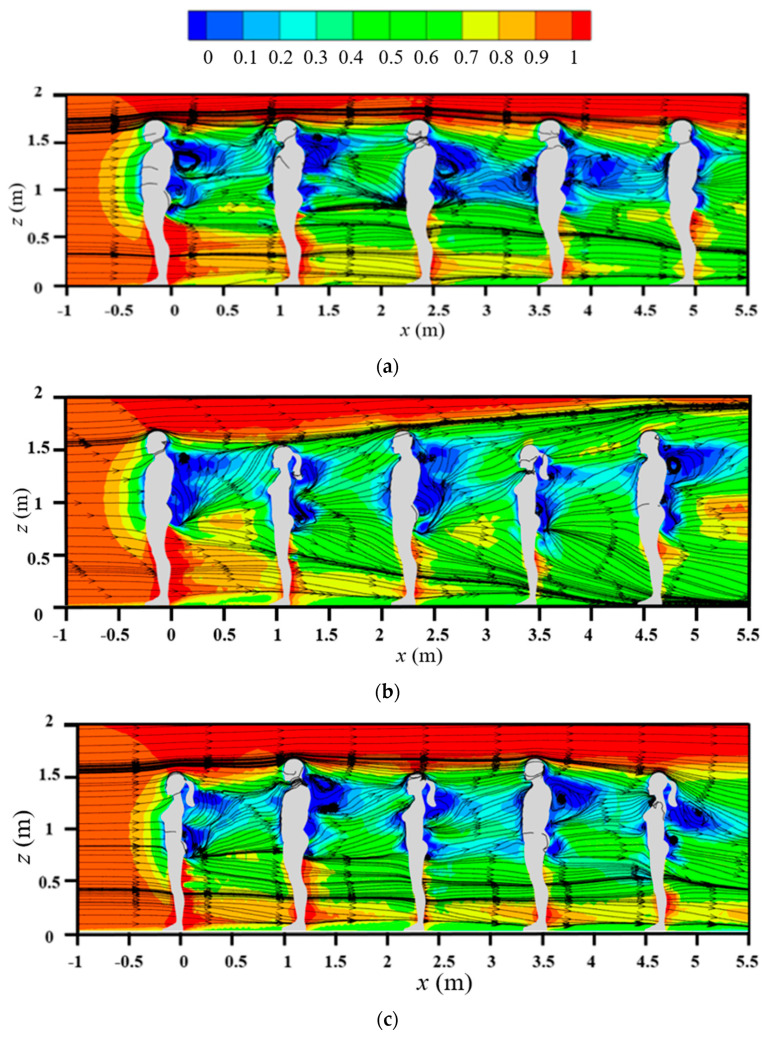
Streamlines and normalized streamwise velocity in the center plane of the domain for three queuing models: (**a**) TQ-1 model; (**b**) TQ-2 model; (**c**) TQ-3 model.

The infection risk was evaluated for five people standing in a queue under an inflow wind condition of *V_in_* = 1.8 m/s; this wind is typical for an outdoor environment. The influence of the body thermal plume on particle dispersion is small when *V_in_* > 0.8 m/s; therefore, it was neglected. However, the evaporation effect was considered. Figure 9 displays the streamlines and normalized streamwise velocity in the center plane of the domain (*y* = 0) for the three queuing models. The overall flow patterns for each queue were similar. Airflow passing the human head was accelerated due to flow separation. Acceleration of the flow through the legs was also discovered and attributed to the Venturi effect. A large reverse-flow region formed behind each body between the hip and head heights; this reverse-flow region was larger behind the male models than the female models because of stronger flow separation. The reverse flow behind the body slowed the downstream diffusion of droplets, trapped the droplets between two bodies, and increased particle deposition on bodies. For the TQ-2 model, the air rose upward slightly after it passed the second and third persons because of the flow disturbance; this could affect the particle dispersion and infection risk for a downstream person.

**Figure 10:**
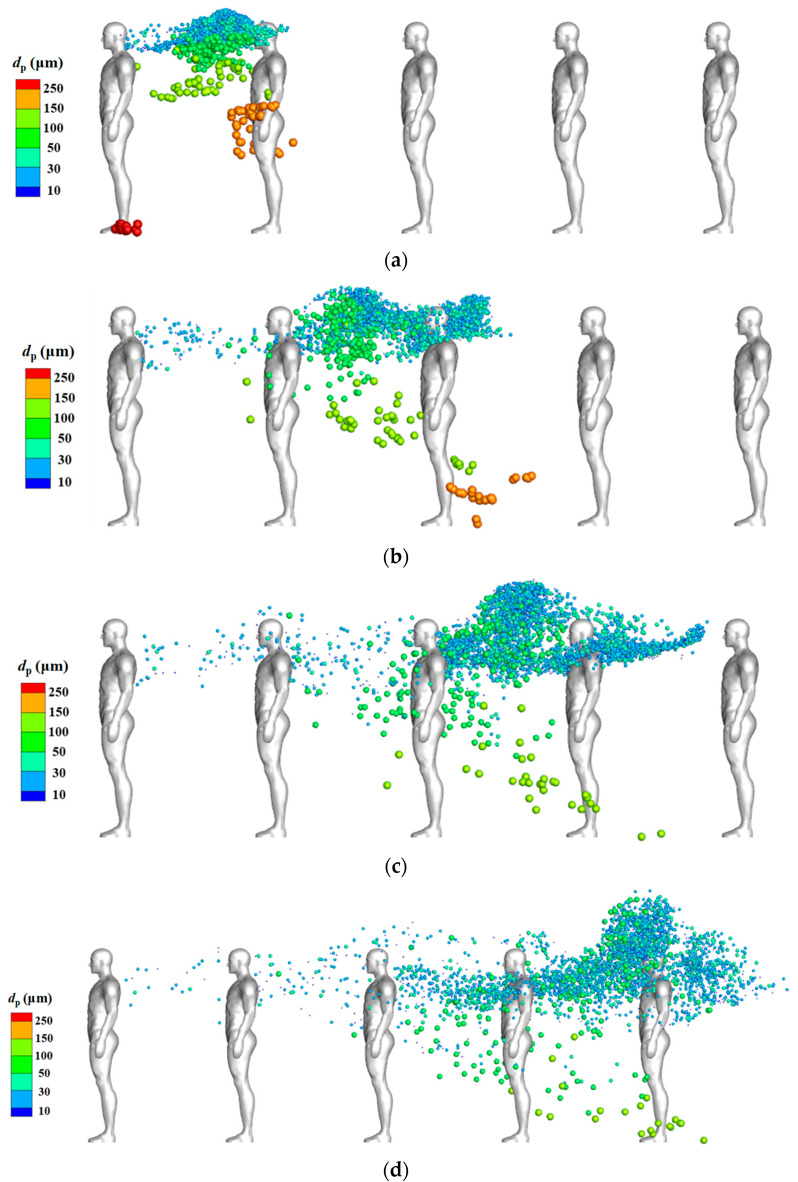
Side view of droplet distribution around virtual manikins for the TQ-1 model at a time of: (**a**) *t* = 1 s; (**b**) *t* = 2 s; (**c**) *t* = 3 s; (**d**) *t* = 4 s.

Figure 10 displays the droplet distributions around manikin models at different time steps for the TQ-1 model. The droplets were coughed into the air from the first person (IP), and the remaining downstream people were SPs. Droplets with different diameters had different dispersion and deposition characteristics. Droplets with an initial diameter of >100 μm gradually fell to the ground due to gravity and did not reach the breathing zone of any SP; however, some were deposited on the SPs. Droplets smaller than 100 μm can reach the breathing zone of downstream SPs, suggesting a substantial exposure risk. Particles with an initial diameter of 75 μm were unlikely to reach the breathing zone of an SP if the evaporation effect was neglected; this could be observed from the suspension height distributions in Figure 8. Some small particles drifted too high above the head; this may also be attributable to the evaporation effect. Overall, the particle dispersion patterns for the TQ-2 and TQ-3 models were largely similar to those for the TQ-1 model. The small droplets traveled slightly higher in the TQ-2 model because of the slight rising flow seen in Figure 9b; these droplets were slightly lower in TQ-3 because the IP was a shorter female model.

**Figure 11:**
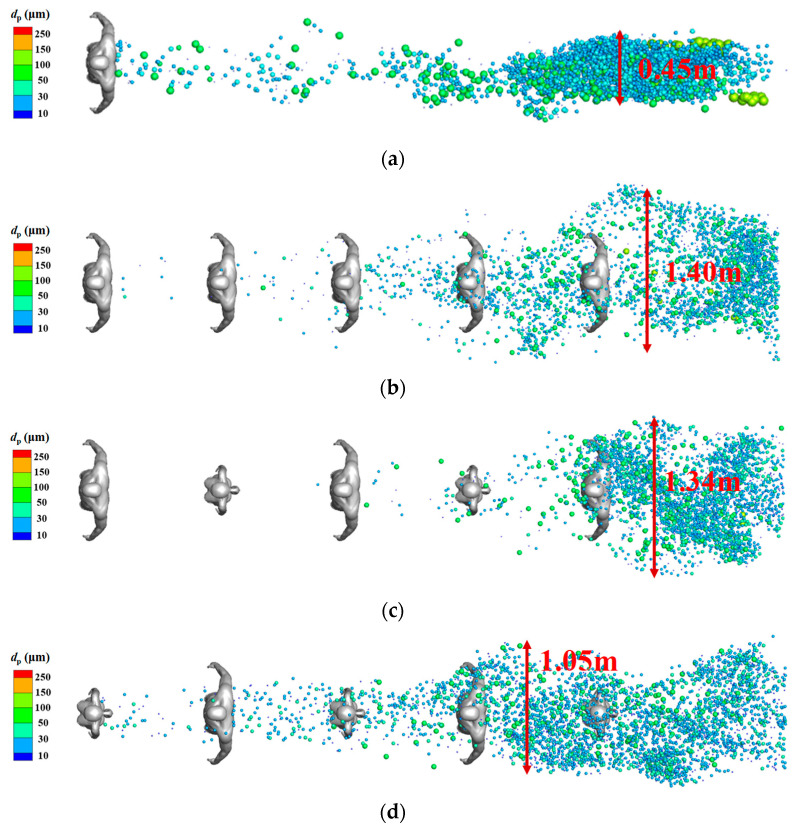
Top view of droplet distributions for isolated and different multi-person models at *t* = 5 s: (**a**) single-person model; (**b**) TQ-1 model; (**c**) TQ-2 model; (**d**) TQ-3 model.

Figure 11b–d display the droplet distribution in the horizontal direction for the TQ-1, TQ-2, and TQ-3 models at *t* = 5 s. The results for the single-manikin model under the same inflow wind condition are also presented for comparison [Figure 11a]. For the single-manikin model, the particles were highly concentrated in a narrow region in the wake of the human body. In the multi-person models, the particles were dispersed much more widely in the horizontal direction because of the flow disturbance by people standing downstream, resulting in a larger exposure area than that in the single-person model. Compared with that in the TQ-3 model, the dispersion areas were wider in the TQ-1 and TQ-2 models because the first person (IP) was a male with a larger projection area in the wind direction, leading to much stronger flow separation. At this instant, many droplets were still trapped among the people in the TQ-3 model.

**Figure 12:**
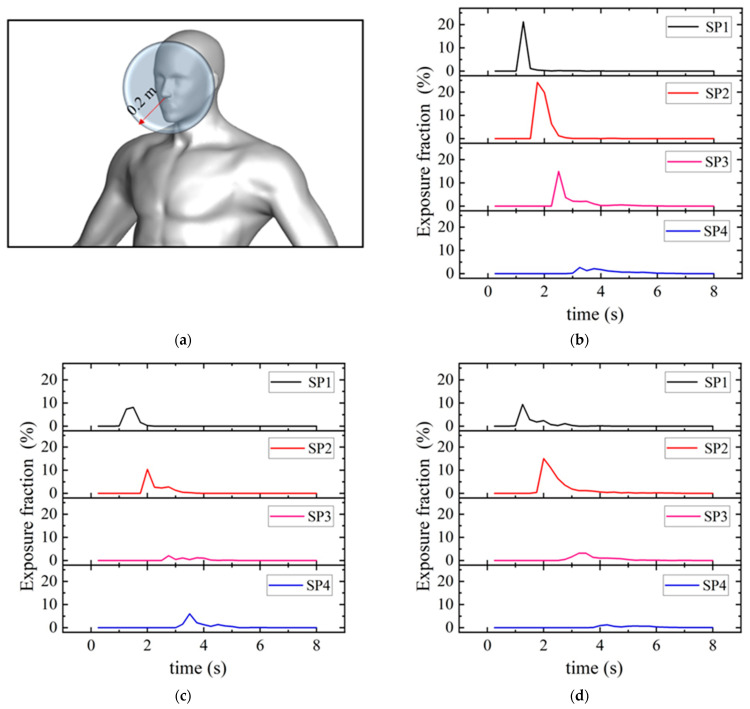
Exposure fraction versus time for each susceptible person in each queuing model: (**a**) definition of the breathing zone; (**b**) TQ-1 model; (**c**) TQ-2 model; (**d**) TQ-3 model.

Several methods can be used to analyze the probability of infection in healthy individuals within different environments, such as the Wells–Riley model [36] and the dose–response model [37]. In this study, the probability of airborne infection was quantitatively evaluated from the exposure fraction in the CFD simulations. The exposure fraction is defined as the percentage of all cough droplets suspended inside the breathing zone of an SP. The breathing zone of an SP was defined as a spherical space with a radius of 0.2 m centered at the nose [Figure 12a]. Virus-carrying droplets suspended in the breathing zone pose the highest infection risk because they can be easily inhaled.

Figure 12b–d present the distribution of exposure fraction over time for each SP in the three queuing models. Overall, the patterns were similar for the three queuing models. The exposure fraction peaked at 1–4 s when numerous small and medium particles simultaneously passed through the breathing zone of an SP; this time was associated with the highest infection risk. The exposure fraction was low for the last person (SP4) in all cases because the preceding people had disturbed the particles, reducing their concentration, and some particles had already deposited on the bodies. The peak value of the exposure fraction was largest in TQ-1 and smallest in TQ-2. This lower value for TQ-2 may have been caused by rising airflow [Figure 9b], which increased the height of the particles relative to the breathing zone.

**Figure 13:**
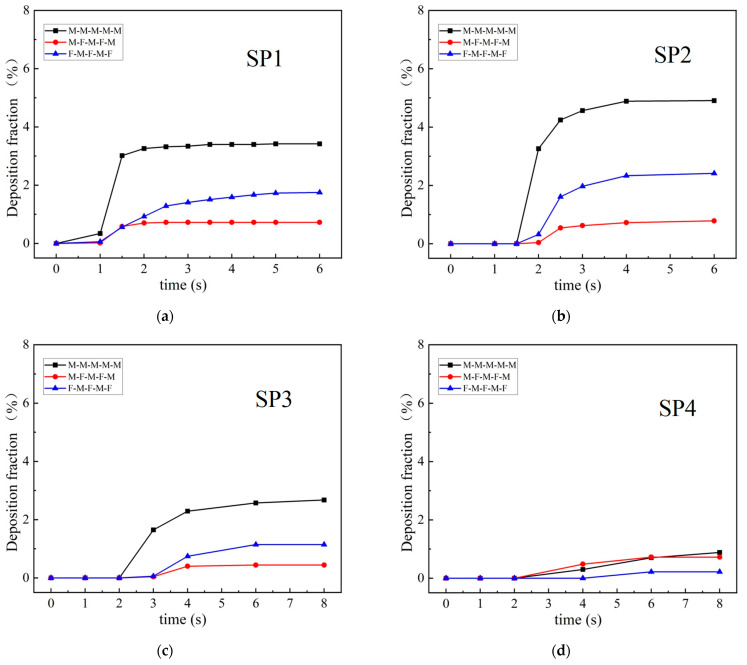
Deposition fractions on the bodies of: (**a**) SP1; (**b**) SP2; (**c**) SP3; (**d**) SP4.

Another indicator for evaluating infection risk is the fraction of deposition on the human body. Figure 13 presents the changes in the fractions of deposition on the body of each SP over time in each queuing model. The deposition patterns were similar for the different queuing modes; the deposition fraction gradually increased and reached a stable maximum after the majority of particles had passed the SP. For each queuing model, the depositions on the first two SPs (SP1 and SP2) were much larger than those on SP3 and SP4; the deposition was lowest for SP4. For all SPs, the final deposition fraction was largest for TQ-1 and lowest in TQ-2, implying that TQ-2 posed a lower infection risk.

**Figure 14:**
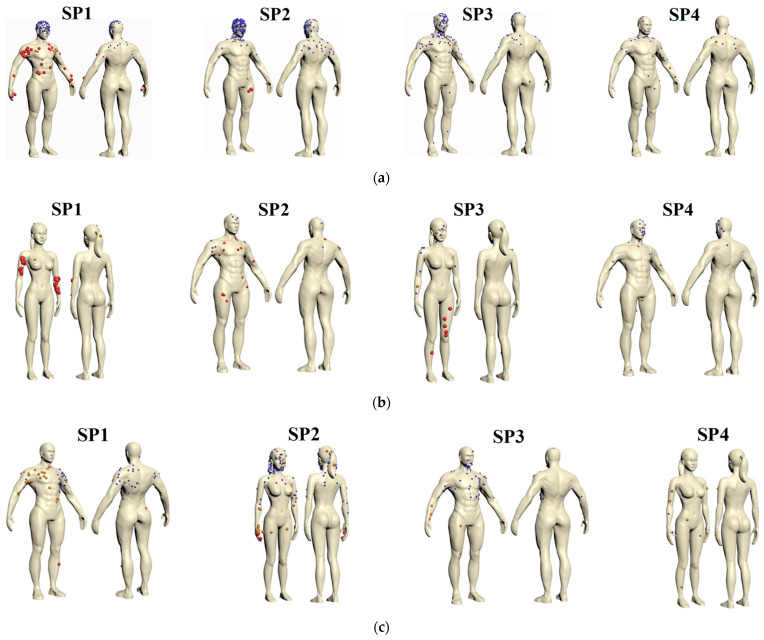
Positions of droplets deposited on the body surface of each SP in three typical queuing models: (**a**) TQ-1; (**b**) TQ-2; (**c**) TQ-3.

However, the deposition fraction had a weaker effect on infection risk than the deposition position did. Figure 14 displays the positions of droplets deposited on the body surface of each SP in the three queuing models. The colors in the figure indicated the particles with different diameters. The deposition numbers displayed in Figure 14 are consistent with the deposition fractions presented in Figure 13. Droplets were deposited on the head, face, back, neck, chest, arms, and legs. Droplets deposited above the chest were mainly small and medium-sized droplets; large droplets were more likely to be deposited below the chest. Numerous droplets deposited on the faces of SP1, SP2, and SP3 in TQ-1, suggesting that SPs queuing in this mode were at a higher infection risk. Fewer particles deposited on the bodies and faces of SPs in queue type TQ-2. In TQ-3, droplets injected from the mouth of the female IP tended to deposit on the chest of the adjacent male and the face of the next female. These depositions on the body could result in a high contact infection risk. Each SP should clean the affected areas on their body to reduce their infection risk.

## 5. Conclusions

In this study, transient CFD simulations were performed to investigate the influences of evaporation and the human body thermal plume on the dispersion of coughed droplets under various inflow wind conditions. The evaporation model adopted in this study was validated through comparison with theoretical results. The flow and particle dispersion around different multi-person models in an outdoor environment were compared. The infection risk was evaluated for each susceptible person in various queuing arrangements by calculating the exposure fraction and deposition fraction. The main conclusions of this study are as follows:

(1) Different-sized droplets exhibit different dispersion patterns when the environmental humidity is different because of evaporation. The duration of evaporation was longer for larger droplets and more humid environments. The dispersion of droplets smaller than 40 μm and larger than 150 μm was insensitive to the environmental humidity. The motion of medium-sized particles was strongly affected by transient evaporation.
(2) The body thermal plume had a large influence on airflow and particle dispersion around the human body under weak inflow wind conditions. When the wind was weak, a clear rising flow and upward drift of small particles were discovered behind the human body; the dispersion of medium-sized particles was affected by both the evaporation and body thermal plume. The body thermal plume also widened the dispersion of droplets in the horizontal direction because it increased turbulence. When the incoming wind was stronger, the influence of the body thermal plume on airflow and particle dispersion became small and was nearly negligible for inflow wind flows larger than 0.8 m/s.
(3) The infection risk was evaluated for people queuing in various arrangements given an outdoor wind speed of 1.8 m/s. Droplets smaller than 100 μm could reach the head of a susceptible person, suggesting a high exposure risk. High exposure fractions and deposition fractions were discovered for only the two people nearest the infected individual. Both the exposure fraction and deposition fraction were the largest for the TQ-1 queuing model and lowest for the TQ-2 queuing model. This suggests that male–female–male–female–male queuing poses a relatively low infection risk in an outdoor environment.

There are some limitations in this study. Although the RNG *k*-*ε* model adopted in this study can accurately predict the airflow around the human body, the result still could be affected by the selection of different turbulence models. The distribution data of cough droplets (such as the number and sizes) used in this study is from Duguid’s measurement in the year 1946. Compared to the new micro-technique and imaging technologies nowadays, the restrictions of experimental conditions may have an impact on measurements. In this study, the infection risk was evaluated by the exposure fraction and deposition fraction of droplets, which means that all coughed droplets were considered to contribute to the infection risk. In fact, not all respiratory droplets can carry infectious viruses. The virus content in droplets needs to be considered in future research to accurately assess the infection risk. This article only studied cough activity in different scenarios, but the other respiratory activities, such as talking and breathing are also significant in delivering virus-containing droplets [5,6,38], which needs to be discussed in future research. Wearing a mask has become a basic prevention behavior to reduce the probability of infection among people, but this study only considered the situation of an open mouth without a mask. It is necessary to assess the effectiveness of wearing a mask in reducing the infection risk in the future.

## Data Availability

The raw data requested can be made to the corresponding author.
